# Editorial: Antimicrobial resistance in zoonotic bacteria

**DOI:** 10.3389/fmicb.2024.1362380

**Published:** 2024-01-23

**Authors:** Guyue Cheng, Liang-xing Fang, Jie Feng, Xunde Li, Zuowei Wu

**Affiliations:** ^1^College of Animal Sciences and Technology, College of Veterinary Medicine, Huazhong Agricultural University, Wuhan, Hubei, China; ^2^College of Veterinary Medicine, South China Agricultural University, Guangzhou, Guangdong, China; ^3^School of Basic Medical Sciences, Lanzhou University, Lanzhou, Gansu, China; ^4^School of Veterinary Medicine, University of California, Davis, Davis, CA, United States; ^5^College of Veterinary Medicine, Iowa State University, Ames, IA, United States

**Keywords:** antimicrobial resistance (AMR), antimicrobial resistance (AMR) genes, zoonotic pathogens, intervention strategies, monitoring and surveillance

Antimicrobial resistance (AMR) is a global issue caused by excessive use of antimicrobial agents in clinical, agricultural, and veterinary settings. This has led to the emergence of resistant microorganisms, threatening public health and food safety. The situation is complicated by a variety of mechanisms in resistance gene transfer and emerging evolution in the genetic makeup of resistant bacteria. Monitoring AMR in wild, companion and farm animals can help combat AMR and limit the spread of antimicrobial resistance genes (ARGs). While several studies have focused on common foodborne pathogens like *Salmonella* and *Campylobacter*, attention must also be given to the role of other zoonotic pathogens in spreading AMR. Effective intervention strategies must be developed to reduce the transmission of ARGs to foodborne and other pathogenic microorganisms.

An understanding of the genetic variation and the transmission mechanisms of important ARGs helps control the propagation of AMR. *Moraxella* spp. cause various diseases in humans and animals and are resistant to many antibiotics, including quinolone, ampicillin, tetracycline, penicillin, and, most importantly, colistin. Che et al. discovered two novel variants of colistin resistance *mcr-1* genes, *mcr-1.35* and *mcr-1.36*, present in *Moraxella* spp. from infected pigs in China. Using a functional cloning assay, they also showed that these genes could transfer resistance from *Moraxella* to *Escherichia coli* DH5α and JM109. In addition to the existence of the *mcr-1* gene, Karim et al. detected the colistin-resistance *mcr-5* gene in Enterobacteriaceae from chicken meat and poultry samples in Malaysia. The *mcr-1* was prevalent in *E. coli* from litter and cloacal swab samples, while *Salmonella* spp. from chicken meat was positive for the *mcr-5*. However, *Klebsiella pneumoniae* isolates were found negative for any *mcr* genes, suggesting further studies on *mcr-5* and its plasmids. The drug-resistance gene *cfr(C)* is known to confer resistance to several critically essential antimicrobials, like streptogramin A, lincosamide, and pleuromutilin, by inducing A2503 methylation in bacterial 23S rRNA. An et al. focused on elucidating the intricate cross-resistance mechanisms of *cfr(C)* in *Campylobacter coli* isolates of swine origin and from the nine *cfr(C)*-positive strains, they identified three novel single nucleotide polymorphism (SNP) sites (674C > A, 19delA, and 890 T > C) for this gene. Five cassettes were present on the chromosome of these six SNP sites, and the remaining one was found on a plasmid-like element. The resistance cluster for aminoglycoside-streptothricin resistance cluster “*aphA3-sat4-aadE*” was associated with three of the six *cfr(C)* SNP variants, while one gene cassette having *pcp* gene (GC-1, GC-4, and GC-5) formed a novel circular intermediate “*pcp*-hp-*cfr(C)-aphA3*”.

Cross-species transfer of AMR in zoonotic pathogens may cause severe public health risks. Guan et al. studied *Wohlfahrtiimonas chitiniclastica* and isolated a novel strain (BM-Y) harboring a *bla*_VEB−1_-gene-carrying plasmid for the first time from a deceased zebra in China. The authors reported that the dissemination of *bla*_VEB−1_ harboring plasmid could enhance resistance, resulting in infections that are difficult to treat. After genomic sequencing, they found three novel insertion sequences (IS), namely IS*Woch1*, IS*Woch2*, and IS*Woch3*, among which IS*Woch1* carried the transcription site of *bla*_VEB−1_ and proved to be an essential promoter for this gene. Card et al. focused on the prevalence of multidrug-resistant (MDR) and clinically significant non-typhoidal *Salmonella* in cloacal samples from migratory birds in Bangladesh. They identified six different serovars of *Salmonella* and an overall prevalence of 13.5%. All strains (MDR) were among these six serovars except the *S*. Perth and *S*. Weltevreden. The authors found that the resistance phenotype was strongly correlated with resistance genes that mainly resided on the *Salmonella* Genomic Islands. Ciprofloxacin-resistant *Salmonella* was associated with mutations in *gyrB* and *parC* genes present on chromosomes, and the *S*. Kentucky belonged to ST198, which is present in both animals and humans, hence a severe public health risk. Abdelhamid and Yousef unveiled the genetic similarities between eggs and poultry-associated *Salmonella enterica* serovar Enteritidis (SE) and indicated that eggs-related SE strains could potentially cause human infections. Comparative genomic analysis of 1,002 SE genomes revealed that SE strains from eggs and poultry have similar genetic lineage but differ from beef-associated SE strains. The *aac(6')-Iaa* and *mdsAB* genes were found to be responsible for prevalent drug resistance, and genetic analyses showed a comparable or similar number of virulence factors in both humans and eggs-associated SE strains.

Zhang et al. suggested the reasonable use of antibiotics according to the drug-resistance genes in clinical settings for patient safety and antibiotic efficacy. They reported that most carbapenem-resistant *Pseudomonas aeruginosa* (CRPA) isolates originated from intensive care unit (ICU) wards and sputum samples. The serious concern was the high-level coexistence of various resistance genes (cephalosporin enzyme, aminoglycoside-modifying enzyme, extended-spectrum β-lactamase, and genes involved in biofilms, membrane channel proteins, I integrons, and efflux systems) and hypervirulence associated genes (*exoS, exoU, exoY*, and *exoT*) in CRPA. The European Union Reference Laboratory for Antimicrobial Resistance (EURL-AR) recommended two reliable methods to separate *E. coli* that produce AmpC, ESBL, and CP enzymes. Hendriksen et al. tested multiple surveillance protocols and found that the Buffered Peptone Water pre-enrichment method was the best, showing the highest sensitivity and specificity for testing minced meat and caecal content samples. Moreover, they described two protocols for isolating and monitoring ESBL- and AmpC-producing *E. coli* and carbapenemase-producing *E. coli* from meat and caecal samples.

Using diet as an intervention to combat AMR has attracted the attention of scientists due to the role of various diets and antibiotics in modulating the composition and diversity of gut and fecal microbiomes. Jinno et al. demonstrated an increase in the relative abundance of beneficial bacteria like Bifidobacteriaceae and Lactobacillaceae in the intestines of enterotoxigenic *E. coli*-infected weaned pigs after supplementation of *Bacillus subtilis* in their diet. Their results also showed that the intestinal microbiome is greatly influenced by diet and other factors, including stress, bacterial infection, and the age of pigs. Similarly, the indispensable role of alkaline arginine in combating the drug-resistant *Salmonella* was studied by Zhu et al. Arginine was found to be an effective adjuvant for aminoglycosides due to its bactericidal effects through increased proton motive force (PMF) and drug uptake by the bacterial cells. During their *in vitro* study, Yang et al. showed that L-leucine can be used as a potential fluoroquinolone adjuvant for fighting clinically resistant *Salmonella* spp. by enhancing the bactericidal effect of sarafloxacin, a unique fluoroquinolone used in veterinary practice. The underpinning mechanisms involved reprogramming resistant bacteria, enhancing metabolism, and increasing ATP, NADH, and reactive oxygen species (ROS) levels inside cells, ultimately promoting antibiotic efficacy.

We need more research on how AMR develops and spreads at the molecular level to understand the mechanisms of AMR and control AMR in zoonotic bacteria from farm and wild animals. We also need enhanced monitoring and improved strategies to fight against AMR and protect public health. This Research Topic presented papers that showed important information and new directions for AMR research.

## Author contributions

GC: Writing – original draft. L-xF: Writing – review & editing. JF: Writing – review & editing. XL: Writing – review & editing. ZW: Writing – review & editing.

